# Mechanisms Underlying the Changes in the Digestive Properties of Chicken Breasts Induced by the Changes in Protein Structure During Frozen Storage

**DOI:** 10.3390/foods14203519

**Published:** 2025-10-16

**Authors:** Xue Bai, Quanyu Zhang, Tingting Zhang, Ying Yu, Xin Du, Xiufang Xia

**Affiliations:** College of Food Science, Northeast Agricultural University, Harbin 150030, Chinayugezuishuai1314@yeah.net (Q.Z.); dbnydxdx@163.com (X.D.)

**Keywords:** chicken breast, frozen storage, digestion, protein structure

## Abstract

This study aimed to investigate the effects of different freezing temperatures and storage durations on the digestive and structural properties of chicken meat proteins. The texture, protein digestibility, particle size, and microstructure of the digested samples were used to characterize their digestive properties. Conformational changes in the digested samples were confirmed by infrared spectroscopy, circular dichroism, and endogenous tryptophan fluorescence spectra. The results revealed that the moisture and protein contents decreased with extended storage and increased temperature. Compared to fresh chicken breasts, protein digestibility decreased as the duration of frozen storage increased. Moreover, the samples stored at −40 °C had higher digestibility than those stored at −18 °C, and the protein structure of the samples stored at −18 °C exhibited more damage and was more likely to aggregate compared to the protein of samples stored at −40 °C. Therefore, a higher freezing temperature and an extended frozen storage duration result in greater structural damage to the protein and lower protein digestibility.

## 1. Introduction

Freezing is a widely utilized method for extending the shelf life of meat during transportation and processing [1]. While frozen storage effectively slows detrimental biochemical reactions in meat, the formation of ice crystals during this process can cause damage to muscle fibers [2]. Meanwhile, the size and distribution of these ice crystals—whether located intracellularly or extracellularly—are influenced by the rate of freezing, while the quantity of ice crystals is governed by the temperature achieved throughout the freezing process [3]. As a result, both the freezing temperature and the duration of frozen storage are critical factors in preserving the sensory quality and structural integrity of meat products.

Throughout the process of frozen storage, the nutrient composition of meat and meat products undergoes significant changes, leading to varying levels of quality deterioration. These changes include alterations in the texture, pH [4], lipid oxidation [5], protein oxidation, aggregation, and crosslinking [1], as well as a reduction in water holding capacity [6]. The formation of ice crystals during freezing, coupled with water migration during thawing and the denaturation and insolubility of proteins can all induce irreversible structural changes in proteins. These changes, in turn, may affect the behavior of proteins during the digestion process [7].

With the enhancement of living standards and the acceleration of modern lifestyles, consumer preferences for meat products have evolved from simply fulfilling hunger to seeking out high-quality options. Simultaneously, there is a rising demand for meat products that are not only nutritious but also safe and hygienic. In the context of international trade, frozen meat has become the predominant form of meat exchange. Consequently, the impact of freezing on the nutritional and digestive properties of meat have become a focal point of research. Feng et al. [8] demonstrated that the digestibility of bighead carp filets protein declined gradually during frozen storage. Wang et al. [9] found long-term high-temperature frozen storage brought about a significant decline in protein digestibility. In recent years, in vitro gastrointestinal (VGI) digestion systems have emerged as valuable tools for studying the physiological effects of food on human health. These systems offer cost-effective, efficient, and ethical alternatives to traditional methods [10].

The previous results of the research group show that frozen storage leads to the formation of large protein aggregates and weakens protein structures [11]; meanwhile, it was also found that the total sulfhydryl content and band intensity of myosin heavy chain and actin were significantly decreased, as the freezing temperature and duration of frozen storage increased, thus damaging the digestive properties of proteins [12]. It has been established in prior studies that the structure of proteins is closely related to the digestive properties of proteins [13]. Structural modifications in proteins, including unfolding, cross-linking, and aggregation, alter their native conformation; this can reduce the accessibility of enzyme cleavage sites and consequently lower protein digestibility. Moreover, during frozen storage, free amino groups decrease as NH_2_ groups are deaminated to carbonyls, accelerating protein oxidation [11]. Due to the disruption of hydrogen bonds and the aggregation of proteins, the secondary structure becomes unstable during storage [14]. In addition, exposure of the hydrophobic core region of proteins leads to disruption of the tertiary structure, promoting intermolecular cross-linking and the formation of oligomers and even large aggregates (e.g., insoluble precipitates). Proteins undergo partial unfolding of secondary and tertiary structures and disruption of non-covalent interactions, such as disulfide and hydrogen bonds, which affects the digestion kinetics of protein digestibility [14].

Frozen storage can alter the structure of proteins, induce protein oxidation, and weaken the digestive properties of proteins [12]. However, the underlying mechanisms connecting changes in protein structure to digestive properties during frozen storage remain poorly understood. Therefore, this study investigated the underlying mechanism by which frozen storage affects protein digestibility in chicken breast. By employing an in vitro gastrointestinal model, we evaluated protein digestive properties and performed correlation analysis with structural changes at the secondary and tertiary levels.

## 2. Materials and Methods

### 2.1. Chemicals

Phosphate-buffer solution, ethanol, pepsin, and trypsin were supplied by Yuanye Biotechnology Co., Ltd. (Shanghai, China). All other chemical reagents were of sequencing grade.

### 2.2. Sample Treatment

Fresh samples (66 pieces of chicken breast, each piece weighing approximately 200 g) were collected from a local market (Harbin, Heilongjiang, China). They were kept in an insulated box with ice packs and taken to the laboratory within one hour, and then the excess fat and connective tissues were removed. Among them, 6 pieces of fresh chicken breast were used as controls and were analyzed directly without freezing storage. The remaining 60 pieces of chicken breast were divided into 2 batches stored at −18 °C (typical frozen storage temperature) and −40 °C (industrial frozen storage temperature). Each batch of 30 pieces was randomly divided into 5 groups and stored for 1, 3, 6, 9, and 12 months (6 pieces per group). Samples from each group were divided into two equal parts, one for hardness and shear force determination (3 pieces) and one for in vitro simulated digestion (3 pieces). Before analysis, samples were thawed at 4 °C for 12 h until the core temperature reached 0–2 °C. The chicken breasts (200.0 ± 5.0 g) were sealed into a cooking bag and put into a pot (Supor, Hangzhou, China) filled with boiling water (100 °C, ratio of meat to water was 1: 3 (*w*/*v*)), and heated for 30 min until the center temperature reached above 75 °C, then cooled to room temperature for further analysis [15].

### 2.3. Determination of Moisture and Protein Content

An oven drying method (AOAC 950.46) for moisture was used and 2 g meat samples were determined by drying them at 105 °C in a constant-temperature oven (HL-3-6DW, KEMAI, Tianjin, China) for 2 h until constant weight was achieved. On the basis of the AOAC (2005) [16] method (AOAC, 2005), a Kjeldahl method (AOAC 928.08) was used to determine the crude protein [17].

### 2.4. Hardness Measurement

Hardness was measured by a texture analyzer (Shanghai Bosin Tech Co., Ltd., Shanghai, China) based on the method of Chatterjee et al. [18] with little modification, and all the samples were cut into 2 × 2 × 2 cm pieces. The texture instrument was equipped with a P/50 probe. The speeds before and after test were 2.0 mm/s and 5.0 mm/s, respectively, with the test interval of 5 s. Data acquisition rate was 400 pps, and the stress variable was 40%.

### 2.5. Shear Force Measurement

The shear force was evaluated using the texture analyzer (Shanghai Bosin Tech Co., Ltd., China) by the research method outlined by Haghighi et al. [19] with slight modifications. The cooked chicken was cut into 2 × 2 × 2 cm slices along the direction parallel to the muscle fibers, and the blade was cut perpendicularly to the muscle fiber direction of the specimen with a cross speed of 2 mm/s.

### 2.6. In Vitro Simulated Digestion

Protein digestibility was evaluated according to the method of Bai et al. [20]. Briefly, for each treatment group, the sample (2 g) was homogenized with 8 mL phosphate buffer (10 mmol/L, pH 7.0) at 10,000 rpm for 2 min in an ice bath. The homogenized sample solution was adjusted to pH 2.0 with 1 M HCl, followed by the addition of pepsin to a final concentration of 6 mg/mL for a 2 h digestion at 37 °C. The reaction was terminated by adjusting the pH to 7.5 with 6 M NaOH to inactivate the enzyme. The resulting pepsin digest (pH 7.5) was then used as the substrate for trypsin digestion, to which trypsin was added to a final concentration of 6 mg/mL, and the mixture was incubated at 37 °C for 2 h. Finally, trypsin digestion was stopped by heating at 90 °C for 5 min. Digestates were precipitated with anhydrous ethanol (1:3, *v*/*v*) at 4 °C for 12 h, centrifuged (10,000× *g*, 20 min), and the dried precipitates were analyzed for protein content to calculate digestibility using the formula:

$$Protein\;digestibility\left(\%\right)=\frac{W_{0}-W_{1}}{W_{0}}\times 100$$

where W_1_ is the protein content (g) in the precipitate after digestion and W_0_ is the protein content (g) in the whole meat before digestion.

### 2.7. Particle Size Measurement

Pre-digestive, gastric, and intestinal digestive fluids were diluted with 10 mM phosphate buffer (pH 7.0) to a concentration of 1 mg/mL, and a light scattering instrument (Mastersizer 3000, Malvern, Worcestershire, UK) was used to determine their particle size. The settings were as follows: the particle type, nonspherical, relative refractive index at 1.54, absorptivity at 0.001, density of 1 g/cm^3^, and water as a dispersing agent.

### 2.8. Microstructure of the Digestive Samples

Chicken breast samples digested by protease (pepsin and trypsin) were subjected to microstructural analysis. Briefly, 5 μL of the sample and 2 μL of Nile blue (aqueous, 0.1% *w*/*w*) were mixed and placed on a microscope slide [21]. The samples were observed under a 40× objective lens using a confocal laser scanning microscope (Leica Microsystems GmBH, Mannheim, Germany). Image acquisition and analysis were performed with the Leica Application Suite X (LAS X 4.4.0) software.

### 2.9. SDS-PAGE

SDS-PAGE of digestion was performed following the method of Bai et al. [20] with slight modifications. The digestion solution was mixed with a certain amount of sample buffer, and the protein concentration was adjusted (about 10 mg/mL) to make the electrophoresis loading solution. The film was stained and decolorized until the strips were clear and then photographed with a gel imager.

### 2.10. Fourier Transform Infrared Spectroscopy

The freeze-dried sample powder (10 mg) was mixed with 200 mg of spectral pure potassium bromide (dried at 105 °C) in quartz mortar. Afterwards, the powder was ground and poured into a vacuum mold to be pressed into thin sheets. Then, the sample slices were placed in an infrared spectrometer (IR200, Nicolet, WI, USA) for analysis, and images from 400 to 4000 cm^−1^ were obtained.

### 2.11. Circular Dichroism Spectra

Circular dichroism spectroscopy (J-1500, Jasco, Tokyo, Japan) was carried out to evaluate the secondary structural changes in digestion solution under various stage with four accumulations in the far UV area (190–260 nm) [22]. Briefly, the concentration of the digestion solution was diluted to 0.2 mg/mL using phosphate-buffer solution and was then placed in a cell with 0.1 mm optical path length. Each sample was recorded in far ultraviolet region of 190–260 nm and accumulated nine scans at a speed of 50 nm/min.

### 2.12. Intrinsic Tryptophan Fluorescence

The intrinsic tryptophan fluorescence of the digested samples was performed following the method of Li et al. [22] with few modifications. The tryptophan fluorescence spectra were recorded from 300 to 400 nm, with an excitation wavelength of 280 nm, and the slit width for the excitation and emission was 5.0 nm, using a fluorescence spectrophotometer (RF-5301, Shimadzu, Kyoto, Japan).

### 2.13. Statistical Analysis

Data processing was carried out using Statistix 8.1 software (Statistix, Inc., Tallahassee, FL, USA). For the statistical mixed model, each sextuplicate was included as a random factor, and the frozen time and temperature were included as the fixed factor. The one-way ANOVA method was used for the analysis of variance, and Duncan’s multiple range was used to compare the differences between mean values. *p* < 0.05 indicated a significant difference in the results. Origin 2024 (Origin Lab, Northampton, MA, USA) was used to generate the figures.

## 3. Results

### 3.1. Moisture and Protein Content

As shown in Table 1, the moisture content of chicken breast decreased with both storage temperature and duration during frozen storage. The moisture content of fresh chicken breast was 75.61%, which decreased by 4.14% and 1.96% (*p* < 0.05) after 12 months of storage at −18 °C and −40 °C, respectively. Furthermore, a comparative analysis of chicken breast stored at different temperatures revealed that, starting from the third month of storage, the moisture content of samples stored at −40 °C was significantly higher than those stored at −18 °C (*p* < 0.05).

The trend in crude protein content aligned with that of moisture content. Compared to fresh chicken breast, the crude protein content decreased by 3.49% and 2.57% after 12 months of storage at −18 °C and −40 °C, respectively. Notably, the protein content was higher in samples stored at −40 °C than at −18 °C, though the difference was not significant (*p* > 0.05) during the first 3 months; it became statistically significant (*p* < 0.05) thereafter.

### 3.2. Hardness and Shear Force

**Figure 1:**
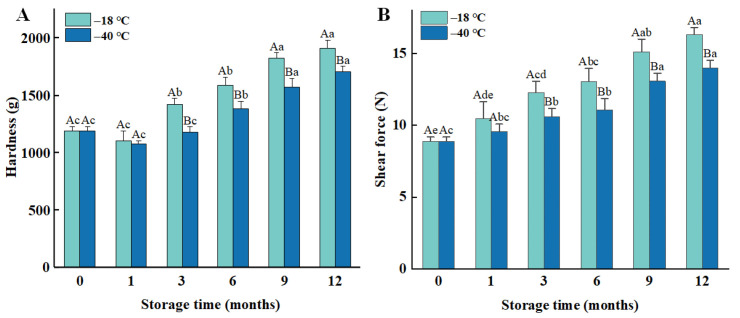
Influence of different frozen temperatures and time periods on hardness (**A**), and shear force (**B**) of cooked chicken breasts. Values represent mean values ± standard deviations of at least triplicate determinations. Different capital letters indicate a significant difference between different temperatures at the same frozen time (*p* < 0.05); different lowercase letters indicated significant differences between different frozen time at the same temperature (*p* < 0.05).

Hardness is one of the key factors affecting meat quality and eating experience, which reflects the firmness of the meat in resisting deformation [23]. As the frozen storage duration increased, the hardness of the cooked chicken meat exhibited a gradual rise (Figure 1A, *p* < 0.05). Specifically, after 12 months of frozen storage at −18 °C, the hardness of the meat increased from 1189 g to 1906 g, and, after 12 months of freezing at −40 °C, it increased from 1189 g to 1704 g. Furthermore, the hardness of the meat stored at −18 °C was higher than that of the meat stored at −40 °C. This indicates that lower freezing temperature and shorter storage duration result in a smaller change in hardness.

Shear force values reflect the tenderness of the internal structure of the meat in resisting breakage, and the results are presented in Figure 1B. These values exhibit a gradual increase with both prolonged storage duration and higher storage temperatures. The shear force of fresh chicken breast was 8.89 N, which increased by 83.69% and 57.14% after 12 months of storage at −18 °C and −40 °C, respectively. Notably, the shear force of the meat stored at −40 °C was lower than that of the meat stored at −18 °C.

### 3.3. In Vitro Digestibility

**Figure 2:**
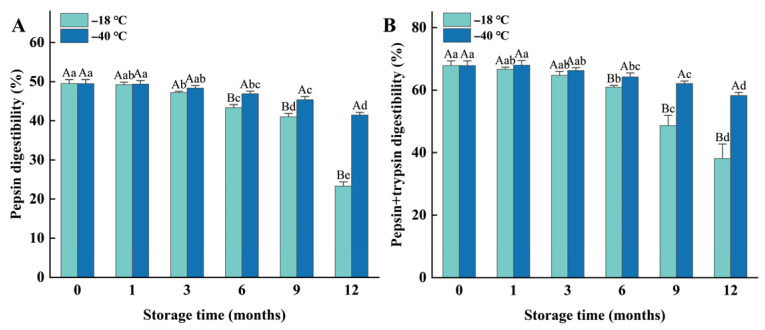
Influence of different frozen temperatures and time periods on the protein pepsin digestibility (**A**) and pepsin + trypsin digestibility (**B**) of cooked chicken breasts. Values represent mean values ± standard deviations of at least triplicate determinations. Different capital letters indicate a significant difference between different temperatures at the same frozen time (*p* < 0.05); different lowercase letters indicated significant differences between a different frozen time at the same temperature (*p* < 0.05).

The in vitro digestibility of meat proteins is considered a highly valuable indicator for evaluating the nutritional quality of meat products [24]. Both pepsin digestibility and pepsin/trypsin digestibility decreased significantly (*p* < 0.05) as the duration of frozen storage increased. Compared to the fresh samples, the pepsin digestibility of the samples stored at −18 °C and −40 °C for 12 months decreased by 26.2% and 8.05% (Figure 2A), respectively, while their pepsin/trypsin digestibility decreased by 29.73% and 9.58% (Figure 2B), respectively. However, no significant differences were observed between the fresh sample and those stored at −18 °C for 1 month (*p* > 0.05), or between the fresh sample and those stored at −40 °C for 3 months (*p* > 0.05). Furthermore, the protein digestibility of the samples frozen at −40 °C was significantly (*p* < 0.05) higher than that of the samples stored at −18 °C after 3 months of storage, indicating that higher freezing temperature and longer storage duration result in lower protein digestibility.

### 3.4. Particle Size

Particle size serves as an indicator of the degree of protein aggregation [14]. The changes in D_4,3_ (equivalent volume–mean diameter) and D_3,2_ (volume–surface mean diameter) are shown in Table 2. The D_4,3_ values of the samples increased significantly (*p* < 0.05) with prolonged frozen storage duration. After 12 months of storage at −18 °C and −40 °C, the D_4,3_ of the pre-digested samples rose by 38.87% and 26.05%, respectively. Similarly, the D_4,3_ of the pepsin-digested samples rose by 109.7% and 98.62%, and the D_4,3_ of the sample digested by pepsin/trypsin rose by 100.6% and 78.90%, respectively. Additionally, significant differences (*p* < 0.05) were observed in particle sizes of the samples before and after digestion. The D_3,2_ values of the digested samples followed a similar trend, showing a significant (*p* < 0.05) increase with an increasing frozen storage duration for both temperatures. After 12 months of storage at −18 °C and −40 °C, the D_3,2_ of the pre-digested samples rose by 69.54% and 28.70%, respectively. Similarly, the D_3,2_ of the pepsin-digested samples rose by 53.50% and 44.97%, respectively, while the D_3,2_ of the samples digested by pepsin/trypsin increased by 60.56% and 47.22%, respectively. Additionally, the D_4,3_ and D_3,2_ values of samples that were kept at −18 °C were consistently larger than those of samples kept at −40 °C for the same duration. This indicates that a higher freezing temperature and longer frozen storage duration result in a larger particle size.

### 3.5. Microstructure of the Digestive Samples

**Figure 3:**
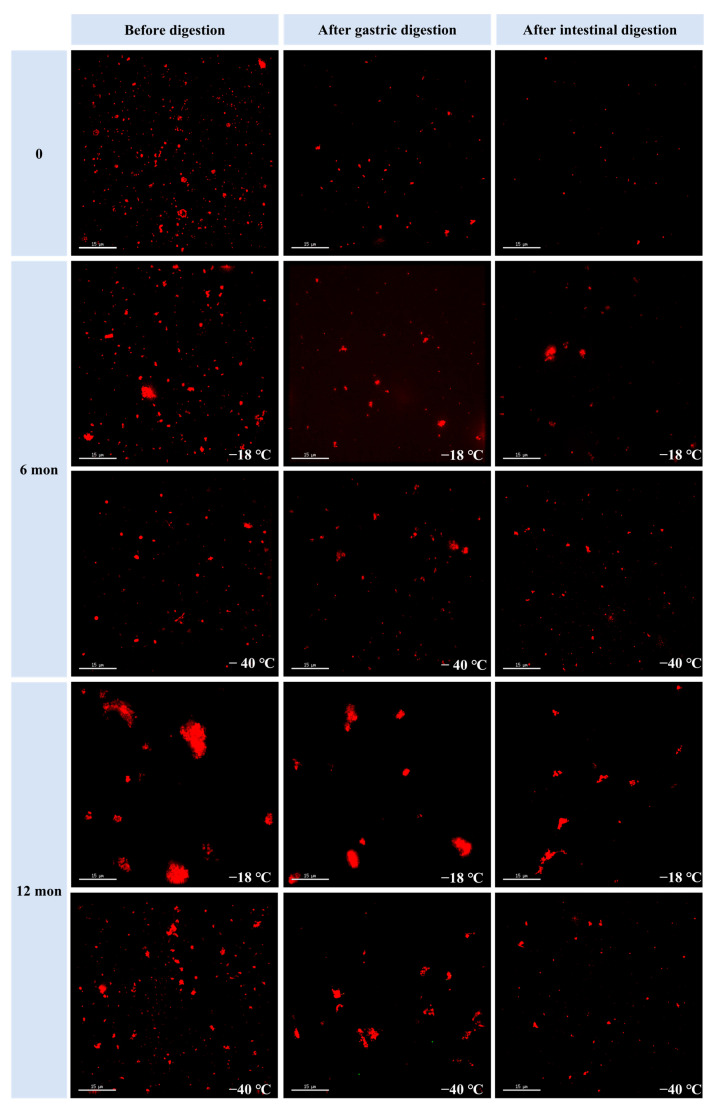
Influence of different frozen temperatures and time periods on the microstructure of cooked chicken breasts before and after simulated digestion.

The effects of freezing temperature and storage duration on the microstructure of the digested samples were studied using confocal laser scanning microscopy. Figure 3 displays the microphotographs of fresh and frozen samples (6 and 12 months) at different digestion stages. The particle size at each digestion stage increased with prolonged frozen storage duration. Additionally, the particle size was consistently larger in samples stored at −18 °C compared to those stored at −40 °C after both 6 and 12 months of storage. These results indicate that higher freezing temperatures and longer frozen storage periods hinder meat digestion by proteases, leading to the formation of larger aggregates in the digested samples. This observation aligns with the trends observed in particle size measurements.

### 3.6. SDS-PAGE

**Figure 4:**
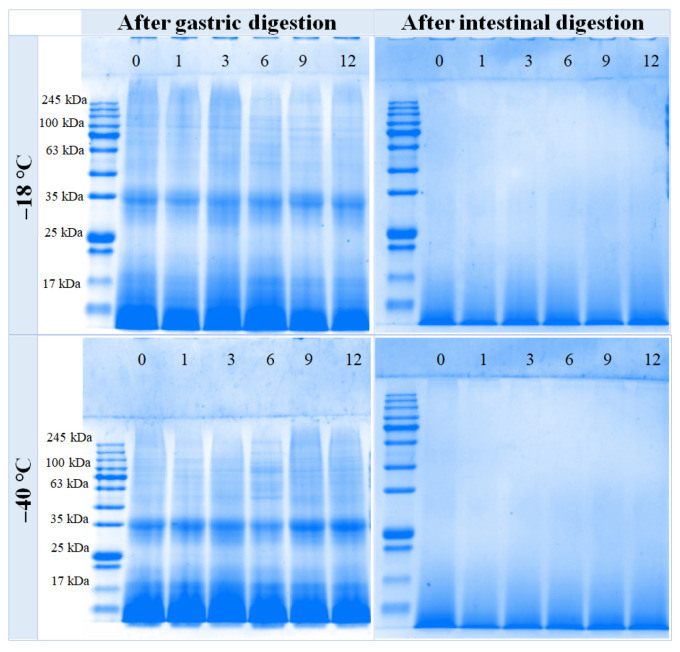
Influence of different frozen temperatures and time periods on the SDS-PAGE of the digestive sample of cooked chicken breast.

The effects of varying freezing temperatures and durations on SDS-PAGE analysis of chicken breast digestive fluid are illustrated in Figure 4. Notably, after digestion by pepsin at −18 °C and −40 °C, the macromolecular bands (~100 kDa) progressively diminished. Distinct bands can be observed near ~35 kDa, with the intensity of these bands increasing as the duration of frozen storage extends. Subsequently, proteins or peptides are gradually degraded into smaller molecular weight bands by pepsin. After further hydrolysis with trypsin, there are essentially no obvious protein bands visible in each sample, only diffuse swimming lanes can be observed on the SDS-PAGE gel of the trypsin digestion products at a molecular weight of less than 17 kDa. The results indicated that protein degradation was more thorough.

### 3.7. FT-IR

**Figure 5:**
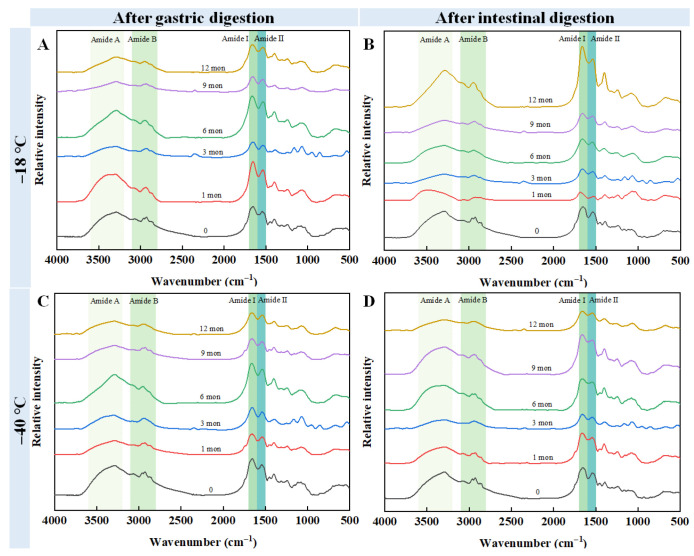
Influence of different frozen temperature and time on the Fourier transform infrared spectra of digestive sample of cooked chicken breast. (**A**) The samples frozen at −18 °C were digested by pepsin; (**B**) The samples frozen at −18 °C were digested by pepsin and trypsin; (**C**) The samples frozen at −40 °C were digested by pepsin; (**D**) The samples frozen at −40 °C were digested by pepsin and trypsin.

To determine the effects of freezing temperature and storage duration on the structural properties of chicken meat digestive fluid, FT-IR analyses were carried out, and the results are presented in Figure 5. After the samples were digested by pepsin, no notable changes were found in the wavenumber of the amide A band of the samples stored at −40 °C. In contrast, the wavenumber of the amide A band for samples stored at −18 °C increased from 3290 cm^−1^ (fresh samples) to 3387 cm^−1^ (12 months of storage). For the fresh samples, the amide I and amide II bands were 1654 cm^−1^ and 1543 cm^−1^, respectively. After 12 months of storage at −18 °C, these bands shifted to 1667 cm^−1^ (amide I) and 1535 cm^−1^ (amide II) (Figure 5A). Similarly, storage at −40 °C resulted in shifts to 1661 cm^−1^ (amide I) and 1537 cm^−1^ (amide II) (Figure 5B). Subsequent digestion with trypsin revealed no notable changes in the wavenumber of the amide A band for samples stored at −40 °C over the same 12-month period. In contrast, for the samples stored at −18 °C for 12 months, the wavenumber of the amide A band exhibited a slight blue shift to 3385 cm^−1^ compared to the fresh sample. The peaks of the amide I and amide II bands for the fresh sample were 1653 cm^−1^ and 1539 cm^−1^, respectively. After 12 months of storage at −18 °C, these bands shifted to 1663 cm^−1^ (amide I) and 1546 cm^−1^ (amide II) (Figure 5C). Similarly, storage at −40 °C resulted in shifts to 1661 cm^−1^ (amide I) and 1543 cm^−1^ (amide II) (Figure 5D). These results suggest that long-term frozen storage induced the partial denaturation of the protein, which resulted in damage to the protein structure.

### 3.8. Circular Dichroism Spectroscopy

**Figure 6:**
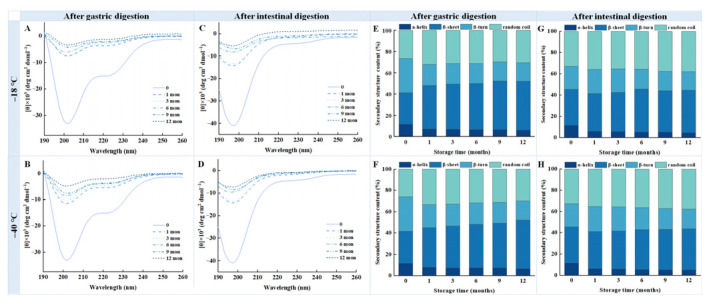
Influence of different frozen temperature and time on circular dichroism spectra and secondary structure contents of digestive samples of cooked chicken breast. (**A**) Circular dichroism spectra of samples digested with pepsin after freezing at −18 °C; (**B**) Circular dichroism spectra of samples digested with pepsin after freezing at −40 °C; (**C**) Circular dichroism spectra of samples digested with pepsin and trypsin after freezing at −18 °C; (**D**) Circular dichroism spectra of samples digested with pepsin and trypsin after freezing at −40 °C; (**E**) Secondary structure contents of samples digested with pepsin after freezing at −18 °C; (**F**) Secondary structure contents of samples digested with pepsin after freezing at −40 °C; (**G**) Secondary structure contents of samples digested with pepsin and trypsin after freezing at −18 °C; (**H**) Secondary structure contents of samples digested with pepsin and trypsin after freezing at −40 °C.

The spectral variations in digested samples under different freezing conditions are illustrated in Figure 6. The absolute values of molar ellipticity at 220 nm for digested chicken breast samples stored at −18 °C and −40 °C exhibited a gradual decline with prolonged storage time. This trend suggests that frozen storage induces the disruption of the protein helix structure, likely due to protein aggregation and protein structure destabilization over extended periods [25]. In addition, two negative peaks at 208 nm and 222 nm can be observed in the far UV region (Figure 6A,B), which are characteristic of an α-helix conformation [14].

As illustrated in Figure 6E,F, after 12 months of storage, the α-helix and β-turn contents of the pepsin-digested samples decreased to 6.5% and 18.3% at −18 °C, and 6.8% and 18.6% at −40 °C, respectively. In contrast, the contents of the β-sheet and random coil increased to 48.2% and 31.5% at −18 °C, and 46.8% and 30.7% at −40 °C, respectively. Similar trends were observed in the samples subjected to further digested by trypsin (Figure 6G,H). Compared to the fresh samples, those samples stored at −18 °C for 12 months exhibited a 6.4% and 2.5% reduction in the α-helix and β-turn content, respectively, whereas the β-sheet and random coil content increased by 9.5% and 8.3%, respectively. Similarly, samples stored at −40 °C for 12 months showed a 5.9% and 1.9% decrease in the α-helix and β-turn content, respectively, alongside a 7% and 7.4% increase in β-sheet and random coil contents. These results indicate that prolonged frozen storage significantly alters the protein structure of the digested chicken breast samples.

### 3.9. Intrinsic Tryptophan Fluorescence

**Figure 7:**
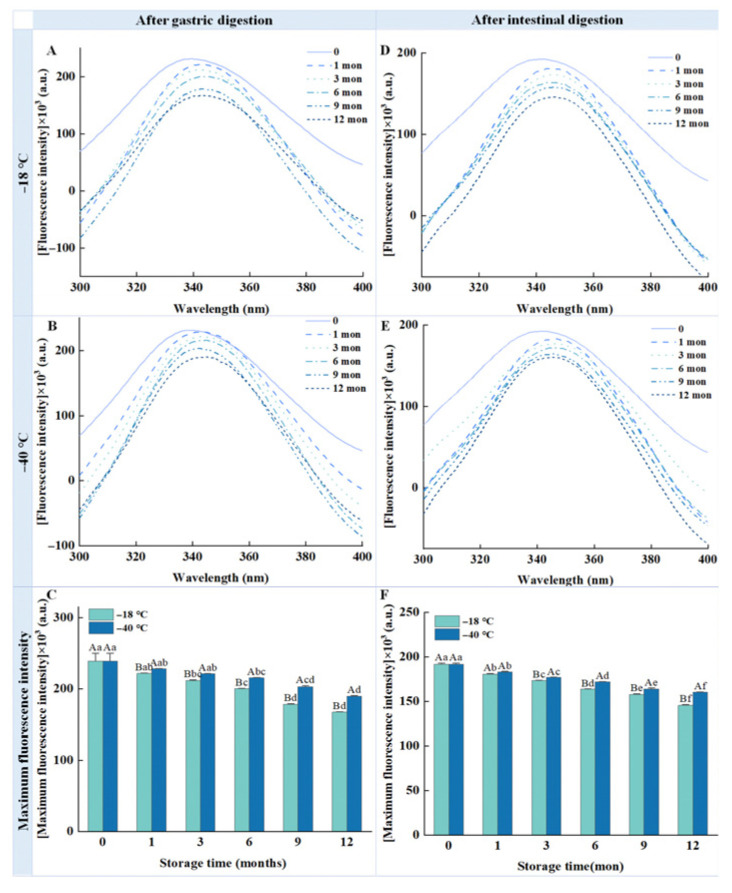
Influence of different frozen temperatures and time periods on the tryptophan intrinsic fluorescence of the digestive samples of cooked chicken breast. (**A**) Tryptophan intrinsic fluorescence of samples digested with pepsin after freezing at −18 °C; (**B**) Tryptophan intrinsic fluorescence of samples digested with pepsin after freezing at −40 °C; (**C**) Maximum fluorescence intensity of samples digested with pepsin after freezing at different frozen temperature; (**D**) Tryptophan intrinsic fluorescence of samples digested with pepsin and trypsin after freezing at −18 °C; (**E**) Tryptophan intrinsic fluorescence of samples digested with pepsin and trypsin after freezing at −40 °C; (**F**) Maximum fluorescence intensity of samples digested with pepsin and trypsin after freezing at different frozen temperature. Values represent mean values ± standard deviations of at least triplicate determinations. Different capital letters indicate a significant difference between different temperatures at the same frozen time (*p* < 0.05); different lowercase letters indicate significant differences between different frozen times at the same temperature (*p* < 0.05).

The structural changes in proteins after long-term frozen storage were studied using tryptophan intrinsic fluorescence, and the results are presented in Figure 7. As the frozen storage time increased, the fluorescence intensity of the chicken breast samples subjected to gastric and intestinal digestion decreased significantly. After 12 months of storage at −18 °C and −40 °C, the fluorescence intensity of the pepsin-digested samples decreased by 27.52% and 17.65% (Figure 7C), respectively, while that of samples digested by pepsin/trypsin decreased by 24.17% and 16.68% (Figure 7F), respectively, compared with the fresh samples. Notably, the fluorescence intensity of the samples digested by pepsin and trypsin after storage at −40 °C was superior to that of the samples kept at −18 °C, indicating that lower temperatures slowed down the rate of protein structural degradation. Furthermore, redshifts were observed in the λmax of the digested samples stored at −18 °C and −40 °C, indicating structural damage to the protein.

### 3.10. Correlation Analysis

**Figure 8:**
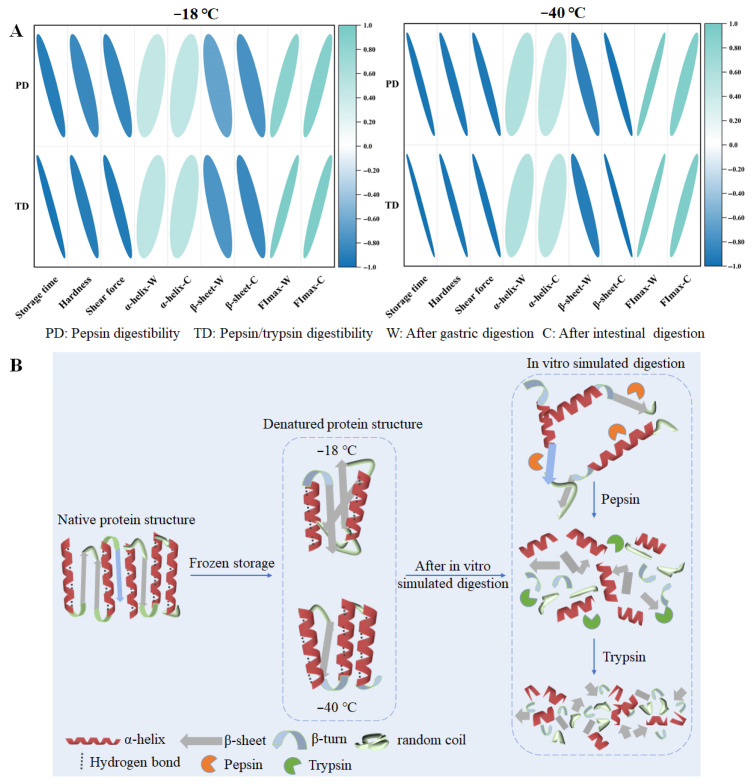
Correlation analysis between textural properties, structural properties, and digestive properties (**A**) and the hypothetical model about how frozen storage affects the protein digestibility of chicken breasts (**B**). The size and color of the circle used to represent the relevant index level could be found. Blue indicates negative values and green indicates positive values.

In order to understand the relationship between textural properties, protein digestibility, and structural properties in chicken breasts more clearly, a correlation analysis was conducted between protein digestibility and various indicators under different storage temperatures. The results are presented in Figure 8A. Regardless of storage temperature (−18 °C or −40 °C), a negative correlation was observed between storage duration and protein digestibility, indicating that prolonged storage reduces protein digestibility to a certain extent. Furthermore, hardness and shear force were significantly negatively correlated with protein digestibility, suggesting that these textural properties could serve as effective sensory indicators for assessing protein digestibility levels. Protein digestibility was negatively correlated with β-sheet content, while it exhibited a positive correlation with α-helix content and FImax.

## 4. Discussion

The moisture and protein content of chicken gradually decreases with the increase in freezing temperature and prolonged storage time. Lower storage temperatures promote faster freezing rates, which facilitates the formation of smaller ice crystals that cause less damage to cellular structures, thereby reducing moisture loss. Meanwhile, extended frozen storage time can lead to protein oxidation and cross-linking, impairing water-holding capacity [26], promoting the loss of soluble proteins. Meanwhile, the denaturation and degradation of proteins during long-term freezing and storage can also lead to a decrease in protein content. This was observed by Hammad Hamed Hammad Mohammed et al. [27], who reported a significant decrease after nine months of frozen storage. Concurrently, both hardness and shear force gradually increased with the extension of temperature and time. Drip loss contributes to meat hardening, and protein cross-linking also leads to texture deterioration by promoting the strengthening of protein structures in muscle tissue, which in turn results in meat toughening [28,29]. Lagerstedt et al. [30] reported a significant reduction in beef tenderness after freezing, a finding consistent with observations by Muela et al. [31] who observed a gradual decline in lamb tenderness over six months of frozen storage—in agreement with the present study. Further supporting these results, Ferreira, Morcuende, Madruga, Silva, and Estvez [32] noted that extended storage elevated meat hardness, associating such textural deterioration with protein cross-linking and structural reinforcement within the muscle matrix. Collectively, these studies underscore that increased hardness in meat is closely linked to protein aggregation [19].

Protein oxidation diminishes digestibility primarily by fostering the formation of enzyme-resistant aggregates via cross-linking and conformational changes [20,21]. These aggregates impede proteolysis through two key mechanisms: first, by sterically shielding cleavage sites from enzymatic access [22], and second, by directly hindering productive binding to the catalytic sites of digestive enzymes [33]. Consequently, the proteolytic accessibility and bioavailability of the protein are significantly reduced.

During gastric digestion, pepsin hydrolyzes polypeptide chains and exposes hydrophobic residues, facilitating subsequent binding and degradation by trypsin into smaller peptides [22]. This proteolytic process is characterized by a progressive reduction in protein particle size, as demonstrated in digested beef by Zhou et al. [34]. Accordingly, in the present study, enzymatically hydrolyzed samples under identical freezing conditions showed a gradual decrease in the size and number of protein aggregates, indicating an increased availability of enzyme contact sites. However, extended frozen storage promoted significant protein aggregation (Table 2), aligning with the findings of Wang et al. [35]. This aggregation is likely driven by freeze-induced protein cross-linking, which increases particle size and sterically hinders protease access to cleavage sites, thereby reducing proteolytic susceptibility [29,36]. The resultant impairment of digestibility with larger aggregate size has been similarly documented in frozen meat by Liu et al. [36] and Pan et al. [12]. Ultimately, freeze-induced denaturation and aggregation diminish the protein’s susceptibility to enzymatic cleavage, preventing its breakdown into smaller peptides [20]. SDS-PAGE suggests that prolonged freezing storage exacerbates protein aggregation, thereby impeding the digestion of proteins by pepsin, which results in a substantial number of peptides and proteins remaining unhydrolyzed.

The amide II band is particularly sensitive to changes in inter- or intramolecular hydrogen bonding, with shifts to higher wavenumbers indicating the breakdown of hydrogen bonds [37]. This is consistent with the results of Jiang et al. [38], who observed that the location of the amide II band of the actomyosin complex moved from 1542 cm^−1^ to 1555 cm^−1^ after 10 d of storage. Such spectral changes may also reflect enhanced hydrophobic interactions resulting from protein oxidation and aggregation during freezing [7]. Furthermore, pepsin can break the peptide bonds of aromatic and hydrophobic amino acids, which results in the exposure of certain groups (e.g., hydrogen bonds), thus, a shift to higher wave numbers is expected [2]. Collectively, these changes indicate that protein structure is damaged during the freezing process, which may subsequently influence protein digestibility.

The α-helix, a key component of a protein’s secondary structure, is stabilized by intra- and inter-hydrogen bonds that maintain normal protein conformation [39]. Hu et al. [40] reported that cryopreservation at ultralow temperatures (−80 °C) caused less protein damage compared to conventional freezing temperatures (−20 °C). Their study also revealed a progressive decrease in α-helix content accompanied by an increase in random coil formation during prolonged frozen storage, which collectively contributed to elevated protein disorder and significant conformational changes. Such conformational changes can hinder protease accessibility to cleavage sites, thereby reducing digestibility.

CD spectroscopy further revealed a structural transition from α-helix to β-sheet or random coil conformations in the digestion products, aligning with the findings of Li et al. [22] in myoglobin samples treated with pepsin and trypsin. Similarly, Zhang et al. [41] observed a gradual decrease in the ratio of positive to negative peaks (Rpn value) in the CD spectrum during digestion, indicating loss of the helix structure of protein and structural loosening. These results support the conclusion that frozen storage induces structural instability in chicken proteins, primarily through aggregation driven by oxidation and denaturation [1]. As freezing temperatures rise and storage duration extends, protein aggregation becomes more pronounced, leading to a greater disruption of the protein secondary structure. Bhat et al. [42] proposed that the protein secondary structure is partially unfolded and hydrogen bonds are broken, which in turn affects protein digestibility. Moreover, Chen et al. [33] demonstrated that most of the digestive properties of the protein during intestinal digestion are positively correlated with structural indicators such as the α-helix. A reduction in α-helix may facilitate structural unfolding, while increased β-sheet content—often associated with aggregation—can hinder proteolysis due to the stability imparted by extensive hydrogen bonding [43]. Bai et al. [13] also reported an inverse relationship between β-sheet content and digestibility. Additionally, Liu, Hu, Wei, and Shi [44] found significant correlations between digestibility and secondary structure components, with α-helix and β-turn negatively affecting digestibility, and β-sheet exhibiting a positive correlation. Taken together, these results underscore that structural integrity—particularly the balance of α-helix and β-sheet structures—plays a critical role in determining protein digestibility, and that frozen storage-induced alterations adversely affect digestive outcomes.

Fluorescence intensity inhibited a gradual decline with the increase in freezing temperature and storage time. This observation aligns with those of Yu et al. [45], who reported a significant reduction in intrinsic tryptophan fluorescence intensity in pork stored under −18 °C with the extension of storage times. The decline in fluorescence intensity is probably due to the oxidative loss of tryptophan residues during frozen storage [46], as well as alterations in their microenvironment—possibly due to the increased exposure of hydrophobic amino acids [25]. Protein aggregation, promoted by partial unfolding and cross-linking under frozen conditions, may also contribute to the diminished fluorescence signal by enhancing steric hindrance and increasing local quenching effects. Furthermore, frozen storage appears to accelerate structural unfolding, shifting tryptophan residues from hydrophobic interiors to a more hydrophilic environment, which may explain the observed redshift in the emission maximum (λmax). These results are consistent with Zhang et al. [47], who attributed similar spectral changes to protein denaturation induced by prolonged freezing. Liu et al. [48] further confirmed that the exposed hydrophobic groups are likely to strengthen protein aggregation through hydrophobic interactions. Such structural alterations can impede enzymatic activity, as exposed hydrophobic domains may hinder peptide access to the catalytic sites of proteases [33]. This is corroborated by Simonetti, Gambacorta, and Perna [49], who identified hydrophobic exposure as a limiting factor for enzymatic hydrolysis. Ultimately, the formation of large, stable protein aggregates reduces proteolytic accessibility, contributing to the decline in digestibility observed in this study.

The schematic diagram of the speculation that frozen storage affects the protein digestibility of chicken breast is shown in Figure 8B. In fresh samples, the protein structure remains its natural state; however, frozen storage causes the formation of large protein aggregates and weakens protein structures [50]. As the freezing rate increased, deterioration reduced. Compared with freezing at –18 °C, freezing at −40 °C reduced oxidation, and delayed the deterioration of protein secondary and tertiary structures during frozen storage [11]. Protein structure destruction is accompanied by a decrease in the content of α -helices and an increase in the random coil structures content [40]. Based on these results, we speculate the structural changes in proteins before digestion after freezing at −18 and −40 °C; compared with the −40 °C frozen storage, the protein structure suffered more serious damage under the −18 °C freezing storage condition, with a decrease in α-helix content and an increase in β-sheet content. These changes resulted in a looser protein structure prone to aggregation. During the in vitro simulated digestion process, the proteins were initially degraded into several peptide chains by pepsin and then continued to be degraded into small fragments or amino acids under the action of trypsin. The formation of ice crystals may disrupt the secondary and tertiary structure of proteins (especially hydrogen bonding and hydrophobic interactions), leading to protein unfolding and exposure of hydrophobic groups, which can further lead to protein aggregation or the formation of insoluble aggregates. These changes may hinder enzyme action and affect subsequent digestion [51]. Additionally, protein denaturation and aggregation during frozen storage reduces the accessibility of pepsin and trypsin to the hydrolysis site, thereby decreasing in vitro protein digestibility.

## 5. Conclusions

In conclusion, the moisture and protein contents of chicken breasts decreased with prolonged storage and increased frozen temperature. This study also demonstrated that the digestibility of chicken meat protein after frozen storage is largely influenced by structural changes. Protein digestibility was reduced with an increasing freezing temperature, and this was negatively correlated with the hardness and shear force of the samples over 12 months. These outcomes indicate that a higher freezing temperature and a prolonged frozen storage duration have adverse effects on protein digestibility. After 12 months, the particle size, β-sheet, and random coil contents of the digested samples that had been stored at −18 °C were greater than those of the samples that had been stored at −40 °C. Consequently, storage at −40 °C appears to mitigate the degradation of protein structure, thereby preserving better digestive properties and structural integrity compared to storage at −18 °C. These findings are expected to provide insights for optimizing the processing and storage conditions of frozen foods and improving their overall quality. Therefore, −40 °C was the appropriate storage temperature for frozen chicken breast in the absence of the consideration of cost. Furthermore, in real-world applications, a graded storage strategy can be adopted: −18 °C for conventional products, −40 °C for high-value-added products or when long-term storage is required.

## Figures and Tables

**Table 1:** Influence of frozen storage temperatures (°C) and time periods (month) on the moisture and protein contents of chicken breast.

Temperature (°C)	Time (Month)	Moisture Content	Protein Content
−18 °C	0	75.61 ± 0.17 ^Aa^	21.33 ± 0.57 ^Aa^
	1	74.72 ± 0.65 ^Ab^	20.89 ± 0.14 ^Aab^
	3	74.94 ± 0.04 ^Bab^	20.27 ± 0.20 ^Abc^
	6	74.54 ± 0.14 ^Bb^	19.87 ± 0.22 ^Bc^
	9	74.14 ± 0.27 ^Bb^	18.95 ± 0.10 ^Bd^
	12	71.47 ± 0.09 ^Bc^	17.84 ± 0.12 ^Be^
−40 °C	0	75.61 ± 0.17 ^Aa^	21.33 ± 0.57 ^Aa^
	1	75.40 ± 0.02 ^Aa^	20.94 ± 0.19 ^Aab^
	3	75.22 ± 0.15 ^Aa^	20.63 ± 0.21 ^Aab^
	6	75.56 ± 0.42 ^Aa^	20.52 ± 0.10 ^Ab^
	9	75.33 ± 0.21 ^Aa^	19.41 ± 0.15 ^Ac^
	12	73.65 ± 0.18 ^Ab^	18.76 ± 0.20 ^Ac^

Values represent mean values ± standard deviations of at least triplicate determinations. Different capital letters indicate significant differences between different temperatures at the same frozen time (*p* < 0.05); different lowercase letters indicate significant differences between different frozen time periods at the same temperature (*p* < 0.05).

**Table 2:** Influence of different frozen temperatures and time periods on the particle size of cooked chicken breast before and after simulated digestion.

Temperature (°C)	Storage Time (Months)	D_4,3_ (μm)			D_3,2_ (μm)		
		Before Digestion	After Gastric Digestion	After Intestinal Digestion	Before Digestion	After Gastric Digestion	After Intestinal Digestion
−18 °C	0	76.98 ± 0.40 ^Ad^	34.69 ± 1.68 ^Ad^	25.54 ± 1.18 ^Ac^	21.67 ± 1.10 ^Ad^	17.28 ± 1.45 ^Ad^	14.40 ± 1.90 ^Ac^
	1	77.57 ± 0.07 ^Ad^	38.12 ± 0.30 ^Ad^	28.92 ± 0.73 ^Ac^	22.69 ± 0.77 ^Ad^	20.79 ± 0.34 ^Ac^	18.73 ± 0.86 ^Ab^
	3	86.41 ± 0.48 ^Ac^	43.49 ± 1.71 ^Ac^	34.98 ± 0.14 ^Ab^	25.08 ± 0.79 ^Acd^	21.67 ± 0.66 ^Abc^	20.21 ± 0.47 ^Aab^
	6	89.05 ± 0.23 ^Ac^	66.43 ± 1.14 ^Ab^	47.84 ± 1.31 ^Aa^	26.32 ± 0.46 ^Ac^	24.19 ± 0.05 ^Aab^	22.22 ± 0.57 ^Aab^
	9	96.31 ± 2.06 ^Ab^	72.29 ± 0.91 ^Aa^	49.31 ± 0.55 ^Aa^	31.54 ± 1.35 ^Ab^	25.93 ± 1.00 ^Aa^	22.77 ± 0.35 ^Aa^
	12	106.9 ± 2.96 ^Aa^	72.75 ± 0.63 ^Aa^	51.23 ± 3.44 ^Aa^	36.74 ± 0.22 ^Aa^	27.06 ± 0.83 ^Aa^	23.12 ± 0.12 ^Aa^
−40 °C	0	76.98 ± 0.40 ^Ad^	34.69 ± 1.68 ^Ae^	25.54 ± 1.18 ^Ad^	21.67 ± 1.10 ^Ac^	17.28 ± 1.45 ^Ad^	14.40 ± 1.90 ^Ac^
	1	76.95 ± 1.10 ^Ad^	35.67 ± 0.57 ^Be^	28.47 ± 0.74 ^Acd^	21.57 ± 0.74 ^Ac^	19.12 ± 0.79 ^Acd^	17.09 ± 0.59 ^Abc^
	3	80.71 ± 1.08 ^Bc^	41.94 ± 0.73 ^Ad^	31.41 ± 0.86 ^Bc^	22.15 ± 0.15 ^Bc^	19.86 ± 0.73 ^Acd^	18.12 ± 0.13 ^Bab^
	6	84.21 ± 0.39 ^Bb^	48.71 ± 0.54 ^Bc^	40.69 ± 0.74 ^Bb^	23.59 ± 0.05 ^Bbc^	21.88 ± 0.11 ^Bbc^	19.43 ± 0.21 ^Bab^
	9	87.09 ± 0.38 ^Bb^	57.52 ± 0.78 ^Bb^	43.92 ± 1.24 ^Bab^	25.65 ± 0.76 ^Bab^	23.93 ± 0.07 ^Bab^	20.34 ± 0.34 ^Bab^
	12	97.03 ± 0.58 ^Ba^	68.22 ± 1.04 ^Ba^	45.69 ± 0.68 ^Ba^	27.89 ± 0.80 ^Ba^	25.05 ± 0.15 ^Ba^	21.20 ± 0.01 ^Ba^

Values represent mean values ± standard deviations of at least triplicate determinations. Different capital letters indicate a significant difference between different temperatures at the same frozen time (*p* < 0.05); different lowercase letters indicate significant differences between different frozen time periods at the same temperature (*p* < 0.05).

## Data Availability

The original contributions presented in this study are included in the article. Further inquiries can be directed to the corresponding author.
